# Factors associated with orofacial myofunctional condition in patients with temporomandibular disorder and somatosensory tinnitus

**DOI:** 10.1590/1678-7765-2025-0515

**Published:** 2026-02-16

**Authors:** Gislaine Aparecida Folha, Nelma Ellen Zamberlan-Amorim, Carina Ferreira Pinheiro-Araujo, Luana Maria Ramos Mendes, Luana Denadai Oliveira Menezes, Gesseany Joice Rodrigues dos Santos, Debora Bevilaqua-Grossi

**Affiliations:** 1 Universidade de São Paulo Faculdade de Medicina de Ribeirão Preto Departamento de Ciências da Saúde Ribeirão Preto São Paulo Brasil Universidade de São Paulo, Faculdade de Medicina de Ribeirão Preto, Departamento de Ciências da Saúde, Ribeirão Preto, São Paulo, Brasil.; 2 Universidade de São Paulo Faculdade de Medicina de Ribeirão Preto Núcleo de Apoio à Pesquisa em Deformidades Craniofaciais Ribeirão Preto São Paulo Brasil Universidade de São Paulo, Faculdade de Medicina de Ribeirão Preto, Núcleo de Apoio à Pesquisa em Deformidades Craniofaciais, Ribeirão Preto, São Paulo, Brasil.

**Keywords:** Tinnitus, Temporomandibular joint disorder, Stomatognathic system, Orofacial pain, Craniofacial pain

## Abstract

Tinnitus is frequently reported in individuals with temporomandibular disorders (TMD), especially in the somatosensory subtype, which can be modulated by afferent input from orofacial and cervical structures. While the impact of orofacial dysfunctions on tinnitus has been explored, the potential reverse relationship remains understudied.

**Objective:**

To investigate the association between somatosensory tinnitus and orofacial myofunctional condition in individuals with TMD.

**Methodology:**

An observational, cross-sectional study was conducted with 47 adults aged 18 to 45 years diagnosed with TMD and self-reported tinnitus. Participants were assessed in a university-based outpatient setting. The primary outcome was the orofacial myofunctional condition, measured using the Orofacial Myofunctional Evaluation with Scores (OMES) protocol. Additional variables included tinnitus intensity and frequency (via acuphenometry), tinnitus-related distress (Tinnitus Handicap Inventory – THI), cervical disability (Neck Disability Index – NDI), craniofacial pain and disability (Craniofacial Pain and Disability Inventory – CF-PDI), and psychological symptoms (Hospital Anxiety and Depression Scale – HADS). Data were analyzed using Spearman correlation and multiple linear regression, and adopted statistical significance was set at p<0.05.

**Results:**

OMES scores showed negative correlations with tinnitus intensity (r=−0.353), THI, NDI, CF-PDI, and age. In the final regression model, only tinnitus intensity remained a predictor of OMES score (β =−0.356; R2=0.127, Confidence Interval −0.20 to −0.02), indicating that greater tinnitus intensity was associated with poorer orofacial myofunctional condition.

**Conclusion:**

These findings suggest that somatosensory tinnitus may negatively impact orofacial motor function in patients with TMD, underscoring the need for integrated and multidisciplinary assessment approaches.

## Graphical abstract

**Figure:**
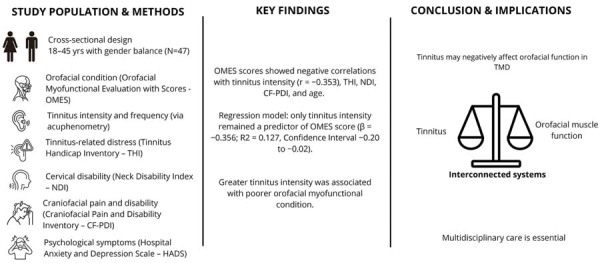

## Introduction

Tinnitus is a frequent and often distressing symptom associated with temporomandibular disorders (TMD), and its presence in individuals with TMD has been well documented in the literature.^1,2^ It is characterized by the perception of a sound or noise in the absence of an external auditory stimulus, often leading to substantial discomfort and psychological burden.^1,3^

Tinnitus may result from dysfunctions in the auditory system (both peripheral and central), the somatosensory system (particularly the cervical and orofacial regions), or their combination. A specific subtype, known as somatosensory tinnitus, occurs when afferent input from the upper cervical spine or temporomandibular region modulates tinnitus perception via neural connections in the brainstem.^1^

The convergence of auditory and somatosensory inputs in the dorsal cochlear nucleus (DCN) has been proposed as a key site for this interaction.^4^ When somatosensory signals excessively activate fusiform cells within the DCN, this heightened activity may exceed the threshold for tinnitus perception, triggering the auditory sensation. These findings support the notion that biomechanical alterations in the orofacial region may play a role in modulating tinnitus perception.^5,6^

A recent meta-analysis confirmed a strong association between TMD and tinnitus, underscoring the clinical importance of exploring their interaction.^7^ Based on this evidence, we hypothesize a possible inverse relationship, that is, tinnitus—especially when somatosensory in origin—may influence orofacial myofunctional status. Patients with tinnitus frequently report broader interconnections between their auditory symptoms, physical pain, and psychological well-being,^3^ all of which may affect the function and control of orofacial structures.

Within this context, orofacial myofunctional evaluation emerges as a valuable tool for investigating how tinnitus and TMD may affect oral motor function. The Orofacial Myofunctional Evaluation with Scores (OMES) protocol is a validated assessment tool designed to characterize appearance and posture, mobility, and orofacial functions in adults with TMD.^6,8^ However, despite its clinical utility, no previous studies have explored the association between OMES scores and clinical features of somatosensory tinnitus in individuals with TMD. While previous research has primarily focused on how temporomandibular and orofacial dysfunctions can exacerbate or modulate tinnitus,^5,6^ no studies have investigated the inverse relationship, that is, whether tinnitus itself, particularly of somatosensory origin, may influence the orofacial myofunctional condition. This represents a critical gap in the literature, as clarifying this bidirectional interaction could provide new insights into the underlying mechanisms linking tinnitus, TMD, and orofacial function, as well as inform multidisciplinary clinical management. Given this scenario, this study intends to contribute to the understanding of the complex interactions among tinnitus, TMD, and orofacial function, with the broader goal of supporting more comprehensive and multidisciplinary therapeutic approaches for this patient population.

Therefore, this study aimed to investigate the relationship between somatosensory tinnitus and clinical variables related to TMD and orofacial myofunctional condition, using the OMES protocol.

## Methodology

### Study design

This cross-sectional study was conducted on patients diagnosed with TMD and somatosensory tinnitus. The presence of TMD was assessed using Axis I of the Diagnostic Criteria for TMD (DC/TMD),^9^ administered by a previously trained physical therapist. The presence of somatosensory tinnitus was confirmed based on the sensitive and specific criteria established by Michiels^10^ (2023), which include the simultaneous onset of tinnitus and pain in the cervical region or jaw, concurrent increases and decreases in both tinnitus and pain, and postural influence on tinnitus intensity. Conversely, the absence of cervical pain or tension in the neck extensor muscles served as valid criteria for the possible exclusion of somatosensory tinnitus.^10^ These questions were presented in informal, non-technical language to ensure clear understanding by the participants, who were assessed individually. Given the cross-sectional design, only one assessment per participant was required.

The study was conducted at a public university hospital, and all data collection occurred over six months (April to September 2024). Participants were invited from the community through social media and local newspapers, and recruiting was based on specific inclusion criteria, which encompassed any type of TMD diagnosis, including painful or articular disorders. Eligible participants were adults of both sexes, aged 18 to 45 years, who self-reported tinnitus (objective or subjective, unilateral or bilateral, and continuous or intermittent).

Exclusion criteria included ongoing physiotherapeutic, orthodontic, or oral rehabilitation treatments; any type of hearing loss or auditory alteration confirmed by audiometry and immittance testing; labyrinthitis; edentulism without the use of prostheses; history of systemic diseases (such as rheumatoid arthritis or fibromyalgia); neurological disorders (e.g., trigeminal neuralgia); history of trauma or surgery; or craniofacial or temporomandibular joint anomalies within the past year.

This study followed ethical guidelines, was submitted to the University’s Research Ethics Committee, and received approval (CAAE number 74181723.0.0000.5440; approval protocol number 6.682.297). All participants signed an informed consent form prior to the commencement of the research, ensuring agreement to participate in assessments and interviews and consent to the processing of personal data.

### Procedures

Participant data were collected using a demographic assessment form that included information on gender, age, weight, height, comorbidities, and self-reported tinnitus.

The diagnosis of somatosensory tinnitus was based on participants’ self-reported information, following the criteria proposed by Michiels^10^ (2023). These criteria indicate a somatosensory origin of tinnitus when there is a temporal relationship between musculoskeletal tension or pain and modulation of tinnitus perception. This approach was adopted considering the nature and objectives of the present study, which focused on analyzing self-reported symptoms and associated factors without performing a standardized clinical assessment. This methodology is consistent with observational and population-based studies in which initial screening for somatosensory tinnitus can be conducted via validated questionnaires and self-perception.

Acuphenometry was performed to determine tinnitus frequency (kHz), intensity (dB), lateralization (unilateral or bilateral), and to characterize the perceived stimulus (white noise, narrowband noise, pure tone, or warble tone). Audiological assessment was conducted in a soundproof booth using an Astera audiometer (Otometrics – Madsen) and HDA200 circumaural headphones.

The exclusion criterion related to hearing loss was defined based on participants’ self-reported information during screening. Participants who reported a previous diagnosis of hearing loss, the use of hearing aids, or a history of otologic diseases associated with auditory alterations were excluded. This criterion ensured that the reported tinnitus was not related to known hearing impairments or other clinical conditions that could interfere with auditory perception. Exclusion was operationalized through a structured questionnaire applied prior to data collection, in which participants confirmed the absence of any diagnosis or treatment for hearing loss.

In addition, a basic audiological investigation was conducted to rule out purely cochlear causes. Pure-tone threshold audiometry was performed at frequencies ranging from 250 to 8000 Hz, as well as high-frequency audiometry from 9000 to 20000 Hz. Hearing was considered normal when thresholds were ≤25 dB HL at all tested frequencies.^11^

To determine air-conduction hearing thresholds, the ascending-descending method was used. If thresholds above 20 dB HL were detected from 500 to 4000 Hz, bone conduction assessment was performed using the B71 bone vibrator to classify the type of hearing loss.^12^

Speech audiometry was conducted using the same equipment as for pure-tone audiometry, including the measurement of the Speech Recognition Threshold (SRT) and Speech Recognition Index (SRI).^13^

The integrity of the tympano-ossicular system was assessed by immittance testing using the Madsen Zodiac 901 (Otometrics), including tympanometry and acoustic reflex testing in both ipsilateral and contralateral pathways.^14^

Subsequently, the participants underwent a comprehensive assessment that included tinnitus impact measured by the Tinnitus Handicap Inventory (THI);^15^ orofacial myofunctional condition assessment using the Orofacial Myofunctional Evaluation with Scores (OMES) protocol, validated for adults with and without TMD^8^; dizziness impact on quality of life assessed via the Dizziness Handicap Inventory (DHI);^16^ pain and disability in patients with TMD with or without other associated pain sources assessed using the Craniofacial Pain and Disability Inventory (CF-PDI),^17^ disability and pain in the neck region assessed using the Neck Disability Index (NDI);^18^ and anxiety and depression symptoms evaluated using the Hospital Anxiety and Depression Scale (HADS).^19^

The questionnaires were administered via structured interviews conducted by speech and language pathologists and physical therapists who had been previously trained. These interviews were supplemented with detailed instructions to ensure consistent and accurate responses. The data from these questionnaires were meticulously collected and recorded, ensuring adherence to standardized protocols for data integrity and reliability. All data were gathered by two specialists and reported in RedCap^Ⓡ^ forms that automatically transferred the information to Excel spreadsheets.

### Tinnitus handicap inventory (THI)

The *Tinnitus Handicap Inventory* (THI)^20^ is a valuable self-report measure for assessing the perceived severity of tinnitus-related issues and their impact on quality of life. It is quick to administer and easy to interpret, containing 25 items. Scores range from 0 to 100, with higher scores indicating greater impact.^14^ This instrument has been validated and adapted for use in Brazil.^15^

### Orofacial myofunctional evaluation with scores protocol (OMES)

This assessment was conducted to characterize orofacial myofunctional condition in terms of appearance and posture, mobility, and orofacial functions. This protocol has been previously validated in Brazil for adult patients with TMD.^8^

Appearance and posture were evaluated by examining the face, lips, tongue, cheeks, hard palate, and maxillomandibular relationship at rest. Mobility was evaluated by observing and assessing movements of the lips, tongue, jaw, and cheeks. Finally, orofacial myofunctional functions were assessed by evaluating respiratory pattern, swallowing, and chewing. It uses ordinal scales of evaluation, allowing both qualitative and quantitative assessments. The sum of scores (TS_OMES) is used to support the diagnosis of orofacial myofunctional disorders and assess their severity; higher TS_OMES scores indicate better orofacial myofunctional condition.^8^

### Dizziness handicap inventory (DHI)

The *Dizziness Handicap Inventory* (DHI) is a tool that assesses the self-perceived effects of dizziness and its impact on quality of life. It contains 25 items and evaluates three domains (functional, emotional, and physical), as well as their repercussions on the patient’s life. Scores range from 0 to 4, with higher scores indicating a greater impact of dizziness on the individual’s life.^21^ The Brazilian version is valid and reliable.^21^

### Hospital anxiety and depression scale (HADS)

The *Hospital Anxiety and Depression Scale* (HADS) is a scale used to assess symptoms of anxiety and depression in individuals with chronic pain, with good sensitivity and specificity for diagnosing these conditions. It consists of 14 items that evaluate anxiety and depression. Each item is scored from 0 to 3, with a maximum score of 21 for each subscale.^22^ The HADS has been validated for Brazilian Portuguese.^19^

### Craniofacial pain and disability inventory (CF-PDI)

This self-administered questionnaire was developed to assess pain and disability in patients with TMD with or without other associated pain sources. It consists of 21 questions, with a total score ranging from 0 to 63. Higher scores indicate worse functional status. The CF-PDI demonstrated acceptable reliability and validity,^23^ including in its version validated for the Brazilian population.^17^

### Neck disability index (NDI)

The NDI is a self-administered questionnaire consisting of 10 questions related to disability and pain in the neck region, assessing cervical disability. Scores range from 0 to 50 points, with higher scores indicating greater disability. The Brazilian version is valid and reliable.^18^

The selection of multiple self-report instruments (THI, DHI, CF-PDI, NDI, HADS) was intentional to provide a multidimensional characterization of participants—including auditory, vestibular, musculoskeletal, and psychological domains—and to explore the complex interactions between tinnitus, TMD, and orofacial myofunctional performance. We acknowledge that administering several questionnaires in a relatively small cohort may have increased participant burden and fatigue, which could potentially influence response accuracy. However, all possible measures were taken to minimize this risk, including completing anamnesis prior to the assessment day, conducting evaluations by experienced professionals to reduce administration time, and systematically offering pauses or breaks whenever needed. Despite these precautions, the possibility of participant fatigue should still be considered when interpreting the findings.

### Statistical analysis

The sample size was estimated based on the assumption that the association between two variables was unidirectional, providing a moderate association coefficient of 0.40, with 80% power and an alpha level of 0.05. The estimations indicated the need for 47 individuals with temporomandibular disorder and tinnitus.

To describe the sociodemographic, anthropometric, and biopsychosocial characteristics obtained from the questionnaires, the following descriptive measures were used: measures of central tendency (mean or median), dispersion (standard deviation), and relative frequency (%). The Kolmogorov-Smirnov test was used to determine whether the data followed a normal distribution.

To explore potential relationships between variables, Spearman correlation and multivariate linear regression analyses were conducted. Bidirectional relationships were estimated using Spearman correlation. Correlations were classified as weak when r < 0.30, moderate when 0.30 < r < 0.60, and strong when r > 0.70. These correlations were used as a preliminary screening step to identify potential predictors for subsequent regression analyses rather than as independent inferential tests. Given their exploratory nature, no correction for multiple comparisons was applied.

To further examine the independent contribution of each variable, multiple linear regression analyses were performed using a backward stepwise procedure. Model comparisons were based on Akaike (AIC) and Bayesian (BIC) information criteria, with lower values indicating better model fit and parsimony. An adequate subject-to-variable ratio was maintained to minimize the risk of model overfitting, following established recommendations of at least 10 subjects per variable.^24^

We report only the models that met all assumptions of multiple linear regression. These assumptions included a linear relationship between the dependent and independent variables, independent and normally distributed residuals, absence of multicollinearity, absence of outliers, and homoscedasticity.

Data were tabulated and analyzed using the JASP software (JASP Team, Version 0.19.3 [Computer software] 2025, Netherlands). The significance level adopted was 0.05.

## Results

### Population characteristics

A total of 47 subjects were consecutively enrolled. The population was characterized by a higher percentage of females (85.11%) than males. The mean age was 30.26 years (+-6,84). See Table 1.

**Table 1:** Descriptive Statistics

N=47	Mean	Standard Deviation
Age (years)	30.26	6.84
BMI (kg/cm^2^)	26.53	4.97
TS_OMES	80.53	6.17
THI score	35.15	23.17
CF-PDI score	23.94	9.73
NDI score	12.72	6.87
DHI score	19.83	19.18
HADS score - Depression	6.57	4.12
HADS score - Anxiety	11.04	4.48
Tinnitus Intensity (dB)	28.76	19.41
Tinnitus Frequency (Hz)	8103.99	5159.16

BMI: body mass index; TS_OMES: Orofacial myofunctional evaluation with scores protocol total score; THI: Tinnitus Handicap Inventory; CF-PDI: Craniofacial Pain and Disability Inventory; NDI: Neck Disability Index; DHI: Dizziness Handicap Inventory; HADS: Hospital Anxiety and Depression Scale.

Factors associated with orofacial myofunctional condition in patients with temporomandibular disorder and somatosensory tinnitus

There was a higher frequency of painful TMD (25/47 or 53.26%) compared to mixed (20/47 or 42.39%) and articular (2/47 or 4.35%) types.

The average THI score was 35 points, indicating that most participants experienced a mild tinnitus impact (18–36) according to the classification of McCombe et al.^25^ (2001). The average DHI score also reflected a mild impact of dizziness (0–30) among the participants. HADS results suggested the presence of anxiety (≥ 8) but the absence of depression (≤ 8) in the sample.

In the orofacial myofunctional evaluation conducted using the OMES protocol, the mean score found (80.53) was 19.57 points below the maximum expected score of 100 points.^8^

Regarding the frequency (in kHz) and intensity (in dB) values obtained in the acuphenometry of all study participants, a higher frequency of bilateral tinnitus (27/47 or 57.45%) was found compared to unilateral tinnitus (20/47 or 42.55%), with one participant also reporting central tinnitus. Individuals with unilateral tinnitus presented higher average values of intensity and frequency than those with bilateral tinnitus.

For unilateral tinnitus, the average frequency was 8927 Hz, with a standard deviation of 5389 Hz and a median of 8000 Hz, ranging from 560 Hz to 16000 Hz. The average intensity was 31 dB, with a standard deviation of 16 dB and a median of 25 dB, ranging from 10 dB to 65 dB.

For bilateral tinnitus, the average frequency was 7273 Hz, with a standard deviation of 4889 Hz and a median of 8000 Hz, ranging from 500 Hz to 16000 Hz. The average intensity was 26 dB, with a standard deviation of 22 dB and a median of 18 dB, ranging from −4 dB to 85 dB.

When asked which acuphenometry stimulus (white noise, narrow band noise, pure tone, or warble) most resembled their tinnitus, the most frequently chosen stimulus was the pure tone (27/47 or 57.89%), followed by narrow band noise (10/47 or 21.05%), white noise (7/47 or 15.79%), and warble (1/47 or 2.63%).

### Analysis

The Spearman correlation analysis between the total score of the OMES protocol and the variables related to somatosensory tinnitus revealed several statistically significant findings, as detailed in Table 2.

**Table 2:** Summary of Spearman's Correlation Results Between TS_OMES and Other Study Variables (n=47).

	Spearman's rho	p-value	Confidence interval	
			**-95%**	**95%**
Tinnitus frequency (Hz)	0.20	0.19	-0.07	0.48
Tinnitus intensity (dB)	-0.35	0.02**	-0.58	-0.08
Age	-0.32	0.03*	-0.57	-0.06
BMI	-0.20	0.19	-0.49	0.06
THI score	-0.29	0.04*	-0.43	0.13
CF-PDI score	-0.29	0.04*	-0.51	0.03
NDI score	-0.38	0.01**	-0.56	-0.04
DHI score	-0.17	0.24	-0.48	0.07
HADS score - Depression	-0.08	0.61	-0.35	0.22
HADS score - Anxiety	-0.28	0.06	-0.54	-0.01

BMI: body mass index; TS_OMES: Orofacial myofunctional evaluation with scores protocol total score; THI: Tinnitus Handicap Inventory; CF-PDI: Craniofacial Pain and Disability Inventory; NDI: Neck Disability Index; DHI: Dizziness Handicap Inventory; HADS: Hospital Anxiety and Depression Scale.

Since no significant correlations were found between TS_OMES and BMI, DHI, or HADS, these variables were not included in the multiple linear regression model.

### Multiple linear regression analysis

A multiple linear regression analysis was performed to identify predictors of orofacial myofunctional condition, as measured by the total OMES score (TS_OMES). The initial model included the four independent variables that had shown significant correlations with TS_OMES in the previous analysis: tinnitus intensity, THI, CF-PDI, and NDI.

As presented in Table 3, the final model demonstrated the best fit (R^2^=0.127; p=0.014), indicating that tinnitus intensity alone explained approximately 12.7% of the variance in OMES scores. Among the tested models, this model presented the lowest AIC and BIC values, indicating the best fit according to information criteria and model parsimony (*R*^2^=0.127). The negative regression coefficient indicates that higher tinnitus intensity is associated with lower OMES scores. Only tinnitus intensity remained statistically significant (β=−0.356; p=0.014), while the other variables (THI, CF-PDI, and NDI) were excluded during the stepwise regression process (Table 3).

**Table 3:** Linear regression models with the total OMES score as dependent variable.

Model parameters	Independent variables	B	SE B	β	t	p	Confidence Interval		Collinearity	
							-95%	.95%	Tolerance	VIF
**Model 1**	Constant	86.19	2.53		34.04	< .001				
R^2^=0.17; Adjusted R^2^=0.09	Tinnitus intensity	-0.09	0.05	-0.27	-1.72	0.09	-0.19	0.02	0.78	1.29
F=2.09; p=0.099	THI score	-0.02	0.04	-0.01	-0.06	0.95	-0.09	0,08	0.82	1.22
AIC/BIC=306.9/318.1	CF-PDI score	-0.07	0.12	-0.12	-0.60	0.55	-0,32	0.18	0.52	1.92
	NDI score	-0.10	0.20	-0.11	-0.51	0.61	-0.50	0.30	0.41	2.43
**Model 2**	Constant	86.15	2.42		35.67	< .001				
R^2^=0.17; Adjusted R^2^=0.11	Tinnitus intensity	-0.09	0.05	-0.28	-1.76	0.09	-0.19	0.01	0.79	1.27
F=2.85; p=0.048	CF-PDI score	-0.07	0.12	-0.12	-0.61	0.54	-0.32	0.17	0.52	1.92
AIC/BIC=305.0/314.2	NDI score	-0.10	0.19	-0.11	-0.55	0.59	-0.48	0.28	0.44	2.27
**Model 3**	Constant	86.23	2.39		36.046	< .001				
R^2^=0.16; Adjusted R^2^=0.12										
F=4.19; p=0.022	Tinnitus intensity	-0.10	0.04	-0.31	-2.18	0.03	-0.19	-7.54x10-3	0.94	1.06
AIC/BIC=303.3/310.7	CF-PDI score	-0.12	0.09	-0.19	0.09	0.19	-0.30	0.06	0.94	1.06
**Model 4**	Constant	83.79	1.53		54.68	< .001				
R^2^=0.13; Adjusted R^2^=0.11										
F=6.53; p=0.014										
AIC/BIC=303.1/308.7	Tinnitus intensity	-0.11	0.04	-0.36	-2.56	0.01	-0.20	-0.02	1.00	1.00

SE: Standard Error; AIC: Akaike Information Criterion; BIC: Bayesian Information Criterion; VIF: Variance Inflation Factor; THI: Tinnitus Handicap Inventory; CF-PDI: Craniofacial Pain and Disability Inventory; NDI: Neck Disability Index.

## Discussion

This study aimed to explore whether tinnitus and other clinical variables associated with TMD could influence orofacial myofunctional status, as assessed by the OMES protocol.^8^ Our findings revealed that tinnitus intensity was inversely correlated with OMES scores, suggesting that greater tinnitus severity may be linked to impaired orofacial neuromuscular performance. This finding supports the exploratory hypothesis that auditory-somatosensory interactions are related to the neuromuscular behavior of orofacial structures.

To the best of our knowledge, this is the first study to explore this association from an inverse and innovative perspective, investigating how the perception of tinnitus, particularly its intensity, may influence myofunctional outcomes across a broad spectrum, encompassing both orofacial and craniofacial domains. Previous research has focused primarily on how orofacial or cervical dysfunctions may modulate or exacerbate tinnitus,^5^ but few studies have explored the potential for tinnitus to act as a modulating variable in orofacial motor control. Our results suggest this possibility, especially in patients presenting with somatosensory tinnitus in the context of TMD.

From a neurophysiological standpoint, the somatosensory pathway is believed to play a key role in modulating tinnitus, particularly when it originates from or is influenced by trigeminal or cervical afferents. When somatosensory input is altered, due to muscle tension, postural compensation, or pain, it may disrupt the normal sensory integration that occurs within the dorsal cochlear nucleus,^4^ contributing to aberrant neural activity associated with tinnitus perception. This mechanism may also impact neuromuscular control of orofacial muscles, particularly if proprioceptive feedback is compromised.

This hypothesis is supported by our findings that lower OMES scores (indicating poorer orofacial myofunctional condition) were associated with higher tinnitus intensity. In the literature, it is well established that patients with TMD present lower OMES scores compared to individuals without this condition (mean 91.32±5.44 vs. 85.64±6.51 in TMD patients).^8^ Although no control group was included in the present study, the values obtained (mean 80.53±6.17), when compared with the previous study, indicate even lower scores in TMD patients, suggesting a greater degree of orofacial myofunctional impairment in our sample. Altered orofacial muscle recruitment, co-activation, and fatigue may arise from sustained muscle tension in response to the discomfort caused by tinnitus. The association between masticatory muscle hyperactivity and somatosensory tinnitus has been previously described,^5,26^ yet our results add a novel layer by suggesting that tinnitus may also impair the functional dynamics of the orofacial system.

Our interpretation is further supported by neuroanatomical models describing how somatosensory tinnitus may originate from afferent inputs to the dorsal root and trigeminal ganglia. These inputs may modulate neural firing patterns within the dorsal cochlear nucleus, ultimately leading to the emergence of tinnitus perception.^27^

Moreover, although other variables such as the Tinnitus Handicap Inventory (THI), Craniofacial Pain and Disability Inventory (CF-PDI), and Neck Disability Index (NDI) were correlated with OMES scores, they did not remain significant in the multivariate regression model. This may suggest that tinnitus intensity has a more direct neurosensory impact on orofacial function than the perceived handicap or regional pain symptoms. Nonetheless, these variables remain clinically relevant, as they may interact in complex ways within the biopsychosocial profile of patients with TMD and tinnitus.

Specifically, there was a moderate and negative correlation between TS_OMES and both tinnitus intensity and age, indicating that greater orofacial myofunctional impairment is associated with higher perceived tinnitus intensity, and that younger individuals tend to present better orofacial myofunctional conditions. This finding supports the hypothesis that orofacial alterations may modulate the perception of tinnitus intensity, as well as the idea that structural or functional changes associated with aging, such as compensatory postural adaptations, may also play a role.

Although weak, a negative correlation was identified between TS_OMES and the Tinnitus Handicap Inventory (THI) and Craniofacial Pain and Disability Inventory (CF-PDI), suggesting that individuals with lower orofacial myofunctional scores tend to report a greater perceived impact of tinnitus on daily life. These findings reinforce the relevance of evaluating craniofacial and orofacial myofunctional condition in individuals with tinnitus and temporomandibular disorders, as dysfunctions in these systems may contribute not only to the modulation of tinnitus perception but also to the amplification of pain-related disability. This highlights the potential benefits of incorporating orofacial myofunctional therapy and physical therapy into multidisciplinary treatment strategies aimed at improving both auditory and musculoskeletal symptoms.

Clinically, this suggests that orofacial myofunctional rehabilitation in patients with TMD and somatosensory tinnitus should extend beyond traditional muscle training. It should include targeted strategies for reducing tinnitus intensity and modulating somatosensory input, such as orofacial myofunctional techniques for muscle relaxation, proprioceptive training, and neuromuscular coordination exercises.^28^

Moreover, educational strategies such as Pain Science Education, recommended in clinical guidelines for the management of chronic painful TMD, may be applicable to individuals with both TMD and tinnitus. This approach involves patient education on key concepts such as variability of pain sensitivity, the potential overprotection driven by pain, the multifactorial contributors to pain, and the biological rationale for strategies aimed at gradually reducing sensitivity. These interventions have been associated with improved disability outcomes in patients receiving manual therapy and exercise-based care for TMD.^29^In addition, an interdisciplinary approach is essential, combining speech-language pathology, physical therapy, dentistry, and psychological support to manage the multifactorial impact of tinnitus and TMD.

We highlight that tinnitus-related discomfort can exacerbate psychological stress, which in turn may amplify muscle hyperactivity and maladaptive postures.^3^ The observed relationship between OMES scores and cervical disability (NDI) reinforces the interconnectedness of the orofacial and cervical systems, highlighting the need for psychological support to address the multifactorial impact of tinnitus and TMD. Thus, cervical stabilization and postural control should also be considered in therapeutic strategies. However, given the small variability (R^2^) found, it is important to consider that other clinical factors may also be related to orofacial myofunctional conditions.

Our findings should be interpreted as exploratory, without implying causality. It seems more plausible that tinnitus intensity functions as an indirect marker of broader biopsychosocial distress rather than as a direct determinant of orofacial motor impairment. This interpretation is consistent with the predominantly mild symptomatology observed in our sample (THI and DHI), the lack of data on TMD duration and severity, and the ceiling effect of OMES scores. Moreover, variables initially correlated with myofunctional performance (THI, NDI, and CF-PDI) did not remain significant in the multivariate model, suggesting overlapping effects potentially influenced by emotional and functional factors that were not fully captured. In this sense, tinnitus intensity may better reflect the subjective burden of pain, stress, and anxiety rather than a direct neuromuscular deficit. Future studies should recruit more heterogeneous samples with respect to TMD severity and duration, adopt longitudinal designs, and explore intervention trials (e.g., orofacial myofunctional rehabilitation) to clarify the causal direction of these associations.

This study presents some limitations. Self-report was used as an operational criterion to identify cases suggestive of somatosensory tinnitus, in accordance with the recommendations by Michiels et al.^10^ (2023), acknowledging the limitations related to the absence of clinical examination while maintaining methodological consistency with the study design.

The predominantly female sample may limit the generalizability of our findings. However, we highlight that, in the general population, women are frequently overrepresented in TMD studies,^8,9^ which may reflect both the higher prevalence of TMD among females and possible sex-related differences in health care-seeking behavior.

The correlation analyses were exploratory and served as a preliminary screening step to identify potential predictors for the regression models. No correction for multiple testing was applied; thus, the possibility of false-positive associations cannot be excluded. Moreover, the regression analyses used a backward stepwise procedure, which—although appropriate for exploratory purposes—may introduce bias, unstable p-values, and model overfitting, particularly in small samples. We mitigated these risks by maintaining an adequate subject-to-variable ratio and comparing models based on AIC and BIC information criteria to ensure parsimony. Finally, the cross-sectional and exploratory nature of the analyses does not allow causal or mediational interpretations; therefore, the observed associations should be interpreted as preliminary and hypothesis-generating.

Additionally, the absence of objective muscular assessments, such as surface electromyography, restricts the understanding of how muscle activation patterns may be altered in this population. Future studies using larger samples, longitudinal designs, and objective neuromuscular data will be essential to further elucidate the bidirectional relationship between somatosensory tinnitus and orofacial motor function.

## Conclusions

The findings of this study indicate that variables related to tinnitus perception and cervical functionality are correlated with orofacial myofunctional dysfunction. Specifically, individuals with higher tinnitus intensity tend to present worse orofacial myofunctional performance, possibly due to neuromuscular or compensatory mechanisms associated with the somatosensory subtype of tinnitus.

These results support the need for an integrated approach to the assessment and treatment of patients with temporomandibular disorders and tinnitus, considering not only the orofacial and joint components but also the potential impact of tinnitus on orofacial motor control.

Although additional studies are needed to deepen the understanding of these associations, particularly across different TMD subtypes and through longitudinal designs that include therapeutic interventions, the current findings are promising. They offer new perspectives on the complexity of orofacial dysfunction and its multifactorial nature, encouraging interdisciplinary dialogue.
